# Identification and functional analysis of novel SLC25A19 variants causing thiamine metabolism dysfunction syndrome 4

**DOI:** 10.1186/s13023-021-02028-4

**Published:** 2021-09-29

**Authors:** Yuanying Chen, Boliang Fang, Xuyun Hu, Ruolan Guo, Jun Guo, Kenan Fang, Jingwen Ni, Wei Li, Suyun Qian, Chanjuan Hao

**Affiliations:** 1 Beijing Key Laboratory for Genetics of Birth Defects, Beijing Pediatric Research Institute, MOE Key Laboratory of Major Diseases in Children, Beijing Children’s Hospital, Capital Medical University, National Center for Children’s Health, Beijing, 100045 China; 2 Pediatric Intensive Care Unit, Beijing Children’s Hospital, Capital Medical University, National Center for Children’s Health, Beijing, 100045 China; 3 Pediatric Intensive Care Unit, Luoyang Maternal and Child Health Hospital, Luoyang, China; 4 Henan Key Laboratory of Pediatric Inherited and Metabolic Diseases, Henan Children’s Hospital, Zhengzhou Hospital of Beijing Children’s Hospital, Zhengzhou, China

**Keywords:** SLC25A19, Thiamine pyrophosphate, Thiamine metabolism dysfunction syndrome 4, Functional study, Exome sequencing, Compound heterozygosity

## Abstract

**Background:**

Thiamine metabolism dysfunction syndrome 4 (THMD4, OMIM #613710) is an autosomal recessive inherited disease caused by the deficiency of *SLC25A19* that encodes the mitochondrial thiamine pyrophosphate (TPP) transporter. This disorder is characterized by bilateral striatal degradation and progressive polyneuropathy with the onset of fever of unknown origin. The limited number of reported cases and lack of functional annotation of related gene variants continue to limit diagnosis.

**Results:**

We report three cases of encephalopathy from two unrelated pedigrees with basal ganglia signal changes after fever of unknown origin. To distinguish this from other types of encephalopathy, such as acute necrotizing encephalopathy, exome sequencing was performed, and four novel heterozygous variations, namely, c.169G>A (p.Ala57Thr), c.383C>T (p.Ala128Val), c.76G>A (p.Gly26Arg), and c.745T>A (p.Phe249Ile), were identified in *SLC25A19*. All variants were confirmed using Sanger sequencing. To determine the pathogenicity of these variants, functional studies were performed. We found that mitochondrial TPP levels were significantly decreased in the presence of *SLC25A19* variants, indicating that TPP transport activities of mutated SLC25A19 proteins were impaired. Thus, combining clinical phenotype, genetic analysis, and functional studies, these variants were deemed as likely pathogenic.

**Conclusions:**

Exome sequencing analysis enables molecular diagnosis as well as provides potential etiology. Further studies will enable the elucidation of SLC25A19 protein function. Our investigation supplied key molecular evidence for the precise diagnosis of and clinical decision-making for a rare disease.

## Introduction

Thiamine metabolism dysfunction syndrome 4 (THMD4, OMIM #613710) is an autosomal recessive inherited disease characterized by bilateral striatal necrosis and progressive polyneuropathy. Patients are usually asymptomatic with normal development until having a febrile illness or viral infection, at which point, the disease rapidly develops and manifests in patients as recurrent episodes of encephalopathy and weakness. The clinical features of patients in the intensive care unit (ICU) are similar to those of acute necrotizing encephalopathy (ANE) [1, 2]. However, unlike ANE, which has a high mortality and disability rate, most patients with THMD4 fully recover, although some patients have mild residual weakness. As there are only a few reported cases, the disease is difficult to clinically identify. However, genetic testing is of great significance for guiding the diagnosis of the disease, clinical treatment, and long-term prognosis.

The disease is caused by a deficiency of *SLC25A19*, which encodes the mitochondrial thiamine pyrophosphate (TPP) transporter [3]. To date, only nine cases (Table 1) have been described worldwide [4–13]. Six variants, namely, c.373G>A (p.Gly125Ser), c.530G>C (p.Gly177Ala), c.576G>C (p.Q192H), c.580T>C (p.Ser194Pro), c.869T>A (p.L290Q), and c.910G>A (p.Glu304Lys), have been reported homozygously in this gene. A variant of *SLC25A19* (c.530G>C, p.Gly125Ser) accounts for Amish lethal microcephaly (MCPHA; OMIM #607196), a disorder with a more severe phenotype [8–10, 14]. The remaining other homozygous variants of *SLC25A19* have been indicated to cause bilateral striatal necrosis with polyneuropathy.

**Table 1:** Reported cases of *SLC25A19* variants

Cases	Variants	Phenotype	References
1	c.373G>A, p.Gly125Ser	Neuropathy and bilateral striatal necrosis	[4]
2	c.495G>A, p.Met165Ile	Thiamine metabolism dysfunction syndrome 4	[5]
3	c.505G>A, p.Glu169Lys	Neuropathy	[6, 7]
4	c.530G>C, p.Gly177Ala	Microcephaly	[8–10]
5	c.580T>C, p.Ser194Pro	Encephalopathy, childhood	[11]
6	c.869T>A, p.Leu290Gln	Neuropathy and bilateral striatal necrosis	[12]
7	c.910G>A, p.Glu304Lys	Neuropathy and bilateral striatal necrosis	[12]
8	~ 4.5 kb incl. partial gene	Thiamine metabolism dysfunction syndrome 4	[5]
9	c.576G>C, p.Gln192His	Bilateral striatal necrosis with polyneuropathy	[13]

Here, we reported the cases of three patients from two unrelated families with encephalopathy associated with fever of unknown origin. All three cases presented with abnormal basal ganglia signals on brain magnetic resonance imaging (MRI). We performed exome sequencing and discovered four novel heterozygous variations, namely, c.169G>A (p.Ala57Thr), c.383C>T (p.Ala128Val), c.76G>A (p.Gly26Arg), and c.745T>A (p.Phe249Ile) in *SLC25A19*. To explain the pathogenicity of these variations, mass spectrometry (MS) was performed. We found that the TPP levels in mitochondria were significantly decreased when carrying the mutated SLC25A19 protein compared with the wild-type protein, indicating that TPP transport activities of mutated SLC25A19 were defective. To the best of our knowledge, this is the first report of *SLC25A19* variants in the Chinese population. Our investigation identified and proved the pathogenicity of novel *SLC25A19* variants, extended the genotype–phenotype spectrum, and guided the clinical diagnosis and decision-making.

## Methods

### Participants

The patients were enrolled from Beijing Children’s Hospital. They were referred to the pediatric ICU (PICU) for severe lethargy, convulsions, or impairment of consciousness. Informed consent was obtained from the parents of the patients. The study was approved by the Institutional Review Board of Beijing Children's Hospital, Capital Medical University (2015–26).

### Exome sequencing, bioinformatics analysis, and Sanger sequencing

Genomic DNA from peripheral blood was extracted, purified, and fragmented into random segments. Genomic DNA was then captured using the Agilent SureSelect Human All Exome V6 Kit (Agilent Technologies, USA), and a sequencing library was prepared. High-throughput sequencing was performed using a HiSeq X Ten sequencer (Illumina, USA), with a reading length of 150 bp. The exome sequencing resulted in > 12 GB of clean data. The average sequencing depth was more than 100×. Sequence alignment was conducted according to the GRCh37/hg19 human reference genome sequence using Burrows–Wheeler Aligner (BWA) and BAM files were created using Picard. Variant calling was performed using Genome Analysis Toolkit (GATK). Variants were annotated and filtered using TGex (https://geneyx.com/geneyxanalysis/). The main reference databases included population databases (dbSNP, 1000G, and gnomAD) and disease databases (Human Gene Mutation Database [HGMD], ClinVar, OMIM, and MalaCards). The pathogenicity of the variants was classified according to the standards and guidelines of the American College of Medical Genetics and Genomics (ACMG) [15]. Primers were designed to amplify the covered exons and flanking regions. The DNA samples of the patients and their parents were amplified using polymerase chain reaction, and Sanger sequencing was performed using ABI 3730xl DNA Analyzer (Applied Biosystems, USA).

### Structure prediction

The protein structure of SLC25A19 was predicted through homology modeling using Phyre2 (http://www.sbg.bio.ic.ac.uk/phyre2). Illustrations of the transmembrane domain of SLC25A19 were drawn based on the prediction using Adobe Illustrator (Adobe, USA).

### Constructs and antibodies

*SLC25A19* was cloned into a pCMV-Tag2B Flag construct using BamHI and HindIII restriction endonucleases. Variations were introduced into the SLC25A19 constructs via site-directed mutagenesis method with slight modifications using KOD-Plus Neo (TOYOBO, Japan) and Dpn I (Thermo Fisher Scientific, USA). The primers used to generate amplicons of SLC25A19 and specified variation sites are presented in Table 2. Flag and β-actin antibodies were purchased from Sigma (Merck, Germany) and an anti-pyruvate dehydrogenase monoclonal antibody was obtained from Abcam (UK).

**Table 2:** Primers used to generate *SLC25A19* constructs and specified variation sites

Primers	Sequences
*SLC25A19*	5′-cgcggatccatggttggctatgacc-3′
	5′-cccaagctttcagcgctggctggct-3′
*SLC25A19* c.169G>A p.A57T	5′-cgcagtgaccccagc**a**caaagtaccatggcatc-3′
	5′-gatgccatggtactttg**t**gctggggtcactgcg-3′
*SLC25A19* c.383C>T p.A128V	5′-gtatgtggtggcctgg**t**tgcctgtatggccac-3′
	5′-gtggccatacaggca**a**ccaggccaccacatac-3′
*SLC25A19* c.76G>A G26R	5′-gctgggtctgtgtct**a**gacttgttactcggg-3′
	5′-cccgagtaacaagtc**t**agacacagacccagc-3′
*SLC25A19* c.745T>A F249I	5′-ctacaggttggaggg**a**ttgagcatgccagag-3′
	5′-ctctggcatgctcaa**t**ccctccaacctgtag-3′
*SLC25A19* c.530G>C p.G177A	5′-aggttttctacaaag**c**cttggctcccacctt-3′
	5′-aaggtgggagccaag**g**ctttgtagaaaacct-3′
*SLC25A19* I33A	5′-gttactcgggcgctg**gcc**agtcccttcgacgt-3′
	5′-acgtcgaagggact**ggc**cagcgcccgagtaac-3′

Mutated sites of each primer are bolded

### Isolation of mitochondria

Mitochondrial fractions were extracted via the differential centrifugation method using the Mitochondria Isolation Kit (MITOISO2, Merck, Germany). Briefly, after transfection with Flag-SLC25A19 and the mutants, HEK 293 cells were harvested via trypsinization. The cells were washed in ice-cold phosphate-buffered saline and centrifuged at 600 × *g* for 5 min. The pellets were resuspended in lysis buffer provided in the kit and incubated for 5 min on ice. The homogenate was centrifuged at 600 × *g* for 10 min to remove the nuclear fraction and unruptured cells. The supernatant was again subjected to centrifugation at 11,000 × *g* for 10 min and the crude mitochondrial fractions were collected. The mitochondrial fractions were resuspended and stored at − 80 ℃ until further use for MS.

### Ms

The mitochondrial extracts and post-mitochondrial supernatants were lyophilized overnight. The samples were then dissolved in a solution of methanol: acetonitrile (1:1, v/v), containing 5% formic acid. The mixture was mixed using a vortex mixer for 5–10 min and centrifuged at 12,000 rpm, 10 ℃ for 5 min. The supernatant was subjected to liquid chromatography-MS analysis. Using Agilent 6545 and Accurate-Mass Q-TOF MS/MS with Agilent 1290 Infinity UHPLC system (Agilent Technologies, Santa Clara, CA, USA), electrospray-ionization liquid chromatography-tandem MS analysis of TPP in positive-ion mode was performed. The mass-to-charge ratio (m/z) for TPP was 425.05. Chromatographic resolution of TPP was achieved using an X Select HSS T3 Column (2.5 μm, 2.1 × 100 mm, Waters Corp., Milford, MA, USA) eluted using a linear gradient from 98% water (containing 20 mM ammonium formate and 0.1% formic acid) (initial phase) to 85% water (containing 20 mM ammonium formate and 0.1% formic acid) and 15% acetonitrile (containing 0.1% formic acid). The mobile phase was then reversed to 98% water. High-performance liquid chromatography flow was 0.4 mL/min. Calibration curves were set at 100–25,000 ng/mL by using standards processed under the same conditions as the samples. The line of best fit was determined by regression analysis of the peak analyte area.

### Statistical analysis

Data were presented as mean ± standard deviation of three independent experiments. The amount of TPP was expressed in terms of nanograms per milligram of protein. One-way analysis of variance was used to conduct statistical analysis, and Dunnett's correction was used to perform multiple comparisons. Significance was set as *P < 0.05, **P < 0.01, and ***P < 0.001.

## Results

### Clinical description

**Fig. 1:**
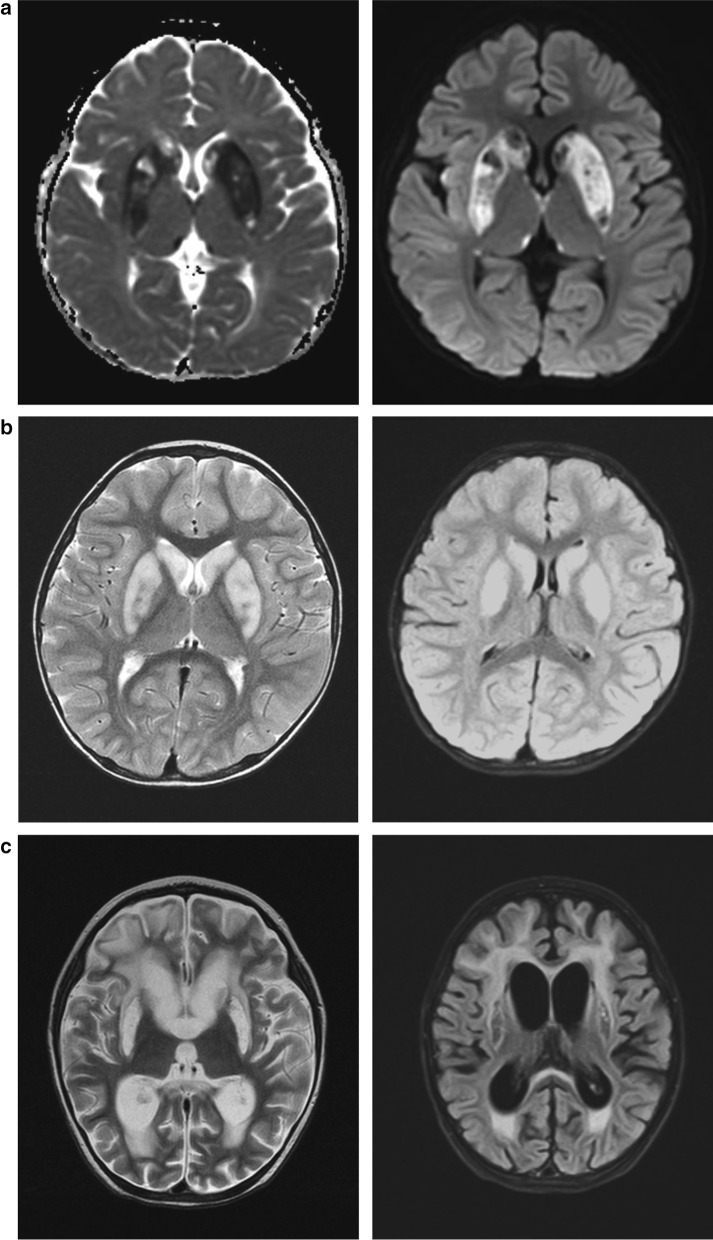
Brain magnetic resonance imaging (MRI) of the three patients. **a** Brain MRI of Patient 1 showing abnormal signals in the bilateral basal ganglia, thalamus margin, hippocampus, midline frontal lobe, and temporal cortex. **b** Brain MRI of Patient 2 displaying abnormalities in the bilateral basal ganglia, thalamus margin, and brainstem. **c** Brain MRI of Patient 3 showing signal changes in basal ganglia and thalamus edge

Patient 1 was a boy aged 4 years and 3 months presenting with normal birth history, growth, and development (height, 115 cm; weight, 18 kg; body surface area, 0.73 m^2^; and head circumference, 50 cm). He showed normal facial features, physical development, and nutrition condition. Nine months after birth, the patient showed somnolence and right upper-limb shaking. Brain MRI showed probable metabolic encephalopathy. Mitochondrial DNA testing was negative. His condition improved after orally taking vitamins B1 and B2, and he had no subsequent developmental delay. At 4 years of age, he was referred to PICU at the Beijing Children’s Hospital for viral infection-induced lethargy, decreased muscle strength and limb tension, diaphragmatic weakness, and expiratory dyspnea; he had to undergo tracheal cannulation and mechanical ventilation for 8 days. Brain MRI showed abnormal signals in the bilateral basal ganglia, thalamus margin, hippocampus, midline frontal lobe, and temporal cortex. Diffusion-weighted imaging was limited (Fig. 1a), and the lactic acid levels in the cerebrospinal fluid (CSF) was normal. Muscle biopsy showed no abnormalities in mitochondrial respiratory chain enzyme activity. Combined with previous medical history, a diagnosis of aggravation of metabolic encephalopathy induced by infection was considered, but the possibility of necrotizing encephalopathy could not be ruled out. During treatment, immunotherapy for necrotizing encephalopathy and vitamin cocktail therapy for metabolic encephalopathy were given along with vitamin B1 (3.33 mg/kg/day) and vitamin B2 (1.67 mg/kg/day). Trio-exome sequencing was performed during his hospitalization. The patient's condition did not deteriorate further; he was transferred to the local hospital PICU for treatment for another 10 days and was taken off the ventilator. Later, the consciousness of the patient cleared and muscle strength recovered; however, low muscle tone, incomplete deep tendon reflex, and extrapyramidal symptoms remained. One-year follow-up showed that the patient survived, exhibiting normal intelligence and behavioral development, but muscle tone and tendon reflex were still decreased.

Patient 2 was a boy aged 2 years and 7 months. He presented with normal birth history and physical and mental development. He had influenza A virus infection-induced febrile illness, became convulsed, and fell into a deep coma. He was admitted to PICU. During the disease, the coma progressively worsened and limb paroxysmal tremors, shaking, and muscle tone increased with passive extension. In the initial stage, the possibility of ANE was suspected. Immunoglobin and methylprednisolone were administered. Vitamin treatment was not given during hospitalization. Brain MRI revealed multiple signal abnormalities in the bilateral basal ganglia, thalamus margin, and brainstem (Fig. 1b). However, the lactic acid levels in the CSF were normal. In the late stage of treatment, he and his parents’ blood were collected for genetic testing in the Center for Medical Genetics of Beijing Children’s Hospital. Four weeks after intubation, he was taken off the invasive ventilator and spontaneous breathing was maintained; however, cough and swallowing reflexes were weak.

Patient 3 was the younger sister of patient 2 aged 1 year and 7 months. She was admitted to local hospital with a deep coma after Influenza A virus infection. She presented many of the same symptoms as her brother, and exome sequencing was performed. Her brain MRI showed pathological changes in the basal ganglia and thalamus edge. Furthermore, the white matter around the bilateral lateral ventricles was demyelinated, the supratentorial ventricles were enlarged, and the sulci were deepened (Fig. 1c). In the recovery process of spontaneous breathing, the clinical symptoms of Patients 2 and 3 were more serious, and they required a longer recovery time for spontaneous breathing than Patient 1. Perhaps, the differences in medical treatment led to the more serious symptoms than those seen for Patient 1. Patients 2 and 3 were discharged from the hospital before obtaining the genetic examination results. Their family members were informed of the results via telephone. They were informed to provide oral vitamin B1 treatment to the patients and followed up at the local Department of Neurology. One month after discharge, Patients 2 and 3 still showed high muscle tone, passive extension of limbs, painful response to external sound stimulation, and ineffectual speak or gaze. At a 3-month follow-up, high muscle tone and passive extension of the limbs had not improved in both patients; they showed a painful reaction to external sound stimulation and were unable to speak but occasionally gazed at the sound source.

### Genetic variation analysis

**Fig. 2:**
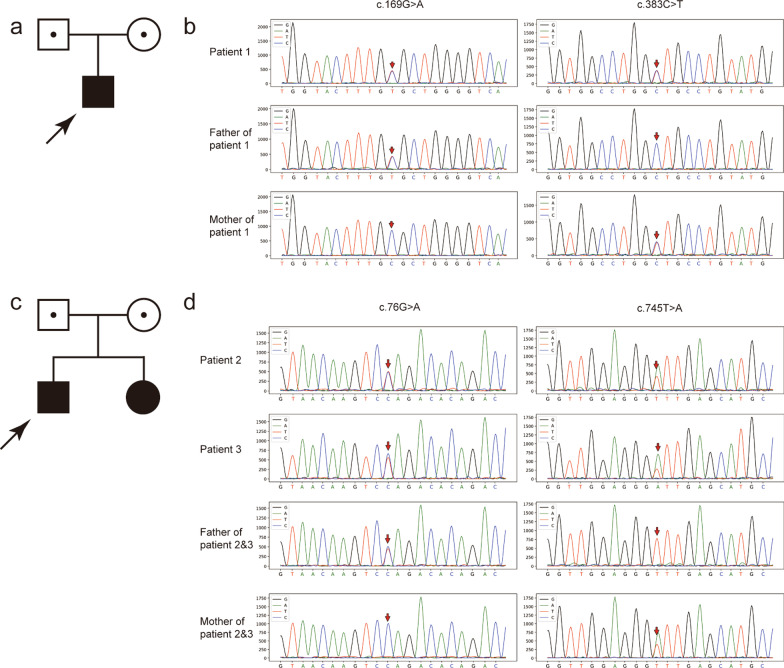
Pedigree and Sanger sequencing of the patients. Pedigree and Sanger sequencing results of Patient 1 (**a**, **b**) and Patients 2 and 3 (**c**, **d**)

**Fig. 3:**
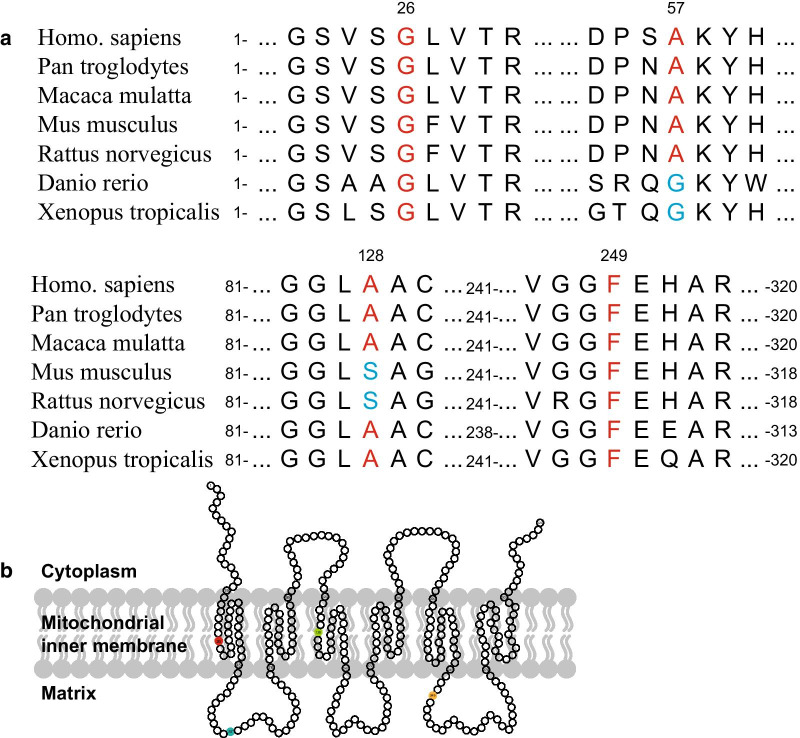
Sequence alignment and predicted topology of SLC25A19. **a** Sequence alignment of *SLC25A19* among species. **b** Predicted topology of SLC25A19 using Phyre2. The circles represent amino acids of SLC25A19, and the variants identified in this study are labeled as red, blue, green, and yellow

Trio-exome sequencing was performed using peripheral blood DNA of all three patients and their respective parents. We identified two novel compound heterozygous variants of *SLC25A19* in Patient 1, c.169G>A (p.Ala57Thr) and c.383C>T (p.Ala128Val), and two compound heterozygous variants in Patients 2 and 3, c.76G>A (p.Gly26Arg) and c.745T>A (p.Phe249Ile). Subsequent Sanger sequencing confirmed that these variations were inherited from their parents (Fig. 2a–d). These variants have not been reported in HGMD and ClinVar and have a low population frequency (Table 3). All variations were predicted to be pathogenic in silico using prediction tools, such as SIFT, Polyphen-2, MutationTaster, and CADD (Table 4). Sequence alignment indicated that the variants were conserved among most species (Fig. 3a).

**Table 3:** Population frequency of *SLC25A19* variants

Patients	Gene	Transcript	Variants		Zygosity	Carrier	gnomAD East Asian	1000 genomes	dbSNP
1	SLC25A19	NM_00112 6122	c.169G>C	p.Ala57Thr	Heterozygous	Father	5.789e−05	–	rs766616256
			c.383C>T	p.Ala128Val	Heterozygous	Mother	–	–	–
2 and 3			c.76G>A	p.Gly26Arg	Heterozygous	Father	0.0005	0.001	rs181826033
			c.745T>A	p.Phe249Ile	Heterozygous	Mother	–	–	–

**Table 4:** Pathogenicity prediction of *SLC25A19* variants

Patients	Gene	Transcript	Variants		SIFT	Plyphen-2	Mutation taster	CADD
1	SLC25A19	NM_00112 6122	c.169G>C	p.Ala57Thr	D	PD	D	D
			c.383C>T	p.Ala128Val	D	PD	D	D
2 and 3			c.76G>A	p.Gly26Arg	D	PD	D	D
			c.745T>A	p.Phe249Ile	D	PD	D	D

### Functional experiments

**Fig. 4:**
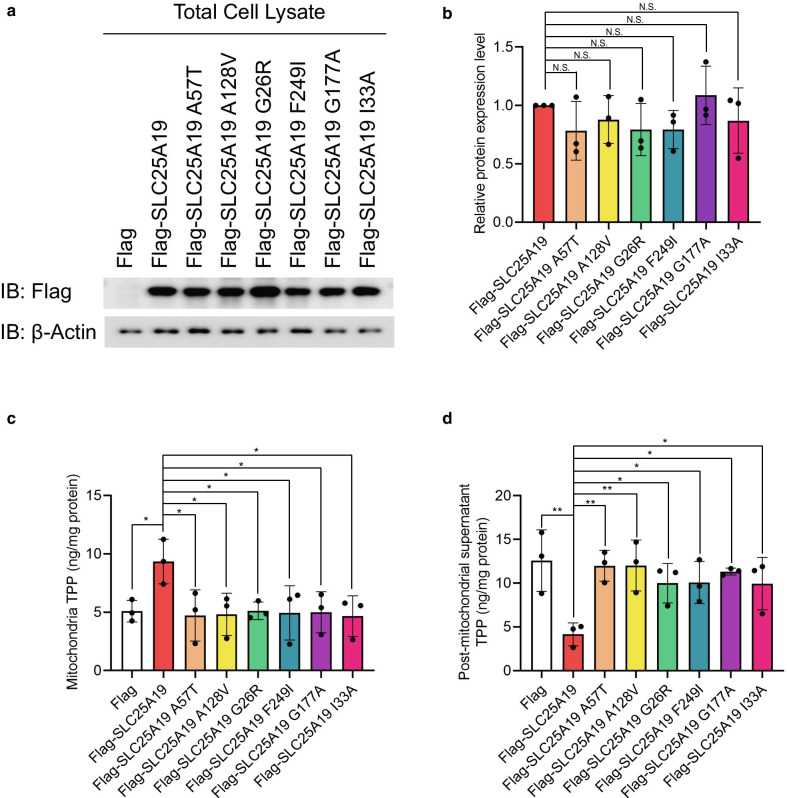
Protein expression and thiamine pyrophosphate (TPP) levels in HEK 293 cells. Protein expression levels of Flag-SLC25A19 and its mutants (**a**, **b**). TPP levels in the mitochondrial fraction and post-mitochondrial supernatant when overexpressed Flag-SLC25A19 and its mutants in HEK 293 cells (**c**, **d**)

Based on Phyre2 analysis, SLC25A19 is predicted to contain six transmembrane domains, and the variation sites are distributed in either the transmembrane helixes or mitochondrial matrix (Fig. 3b). To further confirm the pathogenicity of these variants, functional studies were performed. We cloned *SLC25A19* and its variants into a flag-tagged pCMV-Tag2B vector using site-directed mutagenesis. Two additional SLC25A19 mutants, p.G177A and p.I33A, were used as positive controls, and empty flag-tagged pCMV-Tag2B was used as a negative control. Control plasmids, wild-type, and constructs carrying *SLC25A19* variations were transfected into HEK 293 cells and seeded for mitochondrial isolation. There were no significant differences in the protein expression levels of the wild-type and mutated SLC25A19 protein (Fig. 4a, b). Next, we conducted MS to evaluate TPP levels in isolated mitochondria and post-mitochondrial supernatant to evaluate the thiamine transport abilities of wild-type and mutated SLC25A19 proteins. Mitochondria and post-mitochondrial fractions from HEK 293 cells were isolated after transfection with controls, wild-type SLC25A19, and SLC25A19 mutants. TPP levels were assayed using MS and normalized with total protein. Significantly higher levels of TPP were detected in the mitochondrial fraction of wild-type SLC25A19 than in mutants and controls (Fig. 4c). In contrast, the detected TPP levels in post-mitochondrial supernatant of wild-type SLC25A19 were significantly lower than those of other variants and controls (Fig. 4d). Thus, changes in TPP levels in mitochondria and post-mitochondrial fractions indicated the loss of SLC25A19 transportability in these genetic variants. According to ACMG guidelines, these variants were classified as likely pathogenic (PS3 + PM2 + PP3).

## Discussion

The three patients in this study exhibited viral infection-induced progressive encephalopathy. We conducted exome sequencing and found four novel *SLC25A19* variants, namely, c.169G>A (p.Ala57Thr), c.383C>T (p.Ala128Val), c.76G>A (p.Gly26Arg), and c.745T>A (p.Phe249Ile). Functional studies proved that these variants exhibit defective TPP transportability. Combined with their medical history, MRI imaging results, genetic analysis, functional studies, and follow-up results, we confirmed that these *SLC25A19* variants are likely pathogenic (PS3 + PM2 + PP3) and determined that these patients are likely to have THMD4 or a related condition. Our investigation defined and proved the pathogenicity of these novel *SLC25A19* variants as well as extended our knowledge on the genotype–phenotype characterization of and assisted clinical intervention for patients. Additionally, our investigation demonstrated that definitive molecular diagnosis plays a vital role in predicting long-term prognosis and developing effective drug intervention plans.

SLC25A19 was once considered a mitochondrial deoxyribonucleotide carrier [16]; however, other experiments later demonstrated that SLC25A19 was a TPP transporter [3, 17, 18]. Moreover, it is accepted that *SLC25A19* encodes a transporter that facilitates the movement of TPP across the mitochondrial membrane. TPP is a derivative of thiamine, also known as vitamin B1, and one of the fundamental vitamins present in humans [19]. Mammalian cells obtain this vitamin from their surroundings via transport across the plasma membrane. Several forms of thiamine exist, including free thiamine, thiamine monophosphate (TMP), TPP, and thiamine triphosphate (TTP), in various tissues. Among them, TPP accounts for 80% of the total body thiamine, acting as a cofactor of several complexes of mitochondria and is involved in multiple metabolic processes [20].

Diseases related to *SLC25A19* mainly include MCPHA and THMD4 (Table 1). MCPHA is a severe autosomal recessive metabolic disorder with a poor prognosis. In contrast, THMD4 causes transient neurologic dysfunction, and most patients show complete recovery [4, 12, 13]. According to previous studies, patients with THMD4 are characterized by episodes of encephalopathy in childhood that are often triggered by febrile illness. They usually suffer from encephalopathy, muscular weakness, and the disappearance of deep tendon reflexes. Patients’ brain MRI displays abnormal signals in bilateral basal ganglia, and some patients have high levels of lactic acid in CSF or serums. In some cases, patients recover with mild distal myasthenia or cognitive delay. Treatment with thiamine supplementation at a dose of 400 mg/day can occasionally relieve symptoms.

In our study, all three patients exhibited viral infection-induced encephalopathy and abnormalities in the bilateral basal ganglia after fever. According to the results of genetic testing, the children were highly suspected to have THMD4. Patient 1 fully recovered with normal intelligence and behavior development but still showed decreased tendon reflexes. Patients 2 and 3 had high muscle tone, poor prognosis, and delayed cognitive ability development. In the recovery process of spontaneous breathing, the clinical symptoms of Patients 2 and 3 were more serious compared with Patient 1. The different phenotypes of patients following ventilator removal may indicate the importance of medical treatment and the differences in the genetic variations of SLC25A19. It is reasonable to infer that these variants affect SLC25A19 function through amino acid changes in polarity, charge, and space configuration.

## Conclusions

Three patients developed encephalopathy after viral infection with acute progression in our study. The brain MRI of these patients in the ICU was almost consistent with ANE, which has a high mortality and disability rate. Due to the few case reports on THMD4, it is difficult to clinically identify such cases. Most children with ANE die from respiratory failure, circulatory failure, and severe internal environment disorder due to brain failure within a few days of symptom onset. However, with respiratory and circulatory support, brain protection, and mitochondrial cocktail therapy to get through the acute phase, patients with THMD4 can often survive and have a better neurological prognosis. In this study, we first reported two Chinese nonconsanguineous pedigrees of THMD4. Using exome sequencing, candidate *SLC25A19* was found and unique compound heterozygous variations were identified. Subsequent functional verification confirmed the biological defect of the novel SLC25A19 variants, providing the molecular basis for the clinical diagnosis and treatment. The prognosis of the three reported cases was good; based on these cases, it is suggested that pediatricians pay close attention to the medical history of patients exhibiting an ANE-like phenotype, particularly focusing on the history of the extrapyramidal system and clinical features. In addition, the cooperation of parents with support treatment should be encouraged, informing parents of the importance of continued support to avoid children being affected by viral infection after recovery. Our study assisted in the clinical diagnosis and decision-making of a rare disease.

## Data Availability

The datasets and materials of current study are available from the corresponding author on reasonable request.

## Abbreviations

THMD4: Thiamine metabolism dysfunction syndrome 4
TPP: Thiamine pyrophosphate
MCPHA: Amish lethal microcephaly
PICU: Pediatric intensive care unit
ANE: Acute necrotizing encephalopathy
MRI: Magnetic resonance imaging
MS: Mass spectrometry
HGMD: Human Gene Mutation Database
ACMG: American College of Medical Genetics and Genomics
TMP: Thiamine monophosphate
TTP: Thiamine triphosphate
